# Development and Application of an In Vitro Tick Feeding System to Identify *Ixodes* Tick Environment-Induced Genes of the Lyme Disease Agent, *Borrelia burgdorferi*

**DOI:** 10.3390/pathogens13060487

**Published:** 2024-06-07

**Authors:** Youki Yamasaki, Preeti Singh, Rubikah Vimonish, Massaro Ueti, Troy Bankhead

**Affiliations:** 1 Department of Veterinary Microbiology and Pathology, Washington State University, Pullman, WA 99164, USA; youki.yamasaki@wsu.edu (Y.Y.); preeti.singh@wsu.edu (P.S.); r.kirubananthan@wsu.edu (R.V.); 2 Animal Disease Research Unit, United States Department of Agriculture-Agricultural Research Service (USDA-ARS), Pullman, WA 99164, USA; massaro_ueti@wsu.edu

**Keywords:** Lyme disease, *Borrelia burgdorferi*, in vivo expression technology, *Ixodes* ticks, artificial tick feeding system

## Abstract

The bacterial agent of Lyme disease, *Borrelia burgdorferi*, exists in an enzootic cycle by adapting to dissimilar mammalian and tick environments. The genetic elements necessary for host and vector adaptation are spread across a bacterial genome comprised of a linear chromosome and essential linear and circular plasmids. The promoter trap system, In Vivo Expression Technology (IVET), has been used to identify promoters of *B. burgdorferi* that are transcriptionally active specifically during infection of a murine host. However, an observed infection bottleneck effect in mice prevented the application of this system to study promoters induced in a tick environment. In this study, we adapted a membrane-based in vitro feeding system as a novel method to infect the *Ixodes* spp. vector with *B. burgdorferi*. Once adapted, we performed IVET screens as a proof of principle via an infected bloodmeal on the system. The screen yielded *B. burgdorferi* promoters that are induced during tick infection and verified relative expression levels using qRT-PCR. The results of our study demonstrate the potential of our developed in vitro tick feeding system and IVET systems to gain insight into the adaptive gene expression of the Lyme disease bacteria to the tick vector.

## 1. Introduction

The spirochetal causative agent of Lyme disease, *Borrelia burgdorferi*, is sustained in an enzootic cycle that alternates between a tick vector and a mammalian host. *B. burgdorferi* can be incidentally transmitted to humans via an infected tick bite that potentially leads to lifelong and debilitating clinical symptoms, such as severe arthritis or carditis [1]. Although early antibiotic intervention can successfully clear the infection, some treated individuals continue to have lingering symptoms for six months or longer [1,2,3]. Therefore, developing effective preventative measures to stop the initial infection by *B. burgdorferi* is critical to avoiding the prolonged health effects of Lyme disease. A previous U.S. Food and Drug Administration (FDA) approved recombinant *B. burgdorferi* vaccine for humans took advantage of the host-dependent biology of *B. burgdorferi* by targeting a predominantly presented tick-stage related lipoprotein, outer surface protein A (OspA). Although the vaccine was successful in preventing infection, it was discontinued due to reported adverse effects and poor sales [4]. While there have been additional developments in vaccine development targeting OspA, it is essential to have potential alternatives to prevent the infection and/or disrupt the enzootic life cycle.

To transition between the vastly different physiological conditions of the tick vector and mammalian host, the spirochete alters the expression of genes encoding for protein components necessary for adapting to the respective environments. Prior examinations into *B. burgdorferi* gene expression emulated host conditions in vitro and revealed key regulatory factors such as temperature, pH, mammalian blood, and bacterial density [5,6,7,8,9,10]. Furthermore, DNA microarray analysis and proteomics determined that the bacteria differentially express genetic mechanisms to adapt to different stages of tick colonization: acquisition, persistence, and transmission [11]. These studies provided insight into the mechanisms underlying host and vector adaptation, yet further examination using actual host and tick vectors is needed to identify a more robust scope of the genetic elements necessary for vector-host adaptation by Lyme disease-causing *Borrelia* spirochetes. However, the likelihood is that there are unknown and unrepresented genes involved in the phases of tick colonization that undergo shifts in expression.

In Vivo Expression Technology (IVET) has recently been utilized as a tool for the identification of *B. burgdorferi* genes induced during infection of a murine host [12,13,14]. The IVET system relies on the expectation that a *Borrelia* strain deficient in a gene(s) required for infection will be drastically attenuated in surviving within the host. Pathogen viability can be restored by genetic fusion of sufficiently induced DNA promoters to the necessary gene(s) to complement the genetic deficiency [15,16]. Isolated fusion strains are subsequently screened to distinguish those that are transcriptionally inactive during laboratory cultivation using an in vitro selectable marker. This assay allows for the selection of promoters that are specifically induced in the host environment. 

IVET reporter systems have been utilized to identify murine host-induced genes of *B. burgdorferi* but have yet to be applied to study the tick phase of the enzootic cycle. A past study by our group provided proof of principle for the effectiveness of IVET in *B. burgdorferi*, but a severe infection bottleneck was observed during the experimental challenge [13]. Despite each mouse being inoculated with approximately 500 unique clones, all isolates from each animal were clonal expansions from one dominant clone. This not only limited the utility of IVET for genome-wide promoter screening in mice, but also presented a potential hurdle for our plans to implement IVET screening in ticks because adaptive gene expression likely requires a bloodmeal from a host. The IVET method has been utilized for pathogens harbored by other types of arthropods in past studies [17,18]. 

To overcome this barrier, we adapted a novel silicone membrane-based in vitro tick feeding system to simulate the natural infection of *Ixodes* ticks. Once developed, we utilized this feeding system to feed *Ixodes scapularis* nymphs with *B. burgdorferi* clones harboring our previous IVET plasmid library [13,19,20]. Utilizing this IVET reporter system as a proof of principle, we were able to identify a small list of both novel and previously identified *B. burgdorferi* promoters and their associated genes that are induced in an *I. scapularis* vector.

## 2. Materials and Methods

### 2.1. Adjusting Membrane-Based Artificial Feeding System for Ixodes spp. of Ticks

To account for *Ixodes* tick sensitivity to humidity, a plexiglass and plaster humidity chamber was constructed to enclose the artificial feeding system that was originally described in Vimonish et al., 2020 [21]. Silicone membranes were made using Eco-Flex 00-10 (Smooth-On, Macungie, PA, USA) mixed with Hexane (JT Baker Avantor, Radnor, PA, USA) spread thin across a Goldbeater’s skin (TALAS, Brooklyn, NY, USA). The final membrane thickness for *Ixodes* spp. nymphs reduced to at least 20–40 μm in thickness, measured using a digital caliper. Cotton paper with a one-inch diameter hole in the center was cut to cover the silicone membrane. This was done to reduce the feeding surface area to encourage crowding and provide additional support for the thinner membrane. A plastic *Ixodes* adapter and *Ixodes* receptacle attachment were designed for the more compact concentration of the ticks to further encourage crowding and increasing proximity to the membrane. A wet cotton ball was added to the tick chamber enclosure to ensure relatively high humidity. *I. scapularis* nymphs were provided by BEI Resources, CDC, and Oklahoma State University, Stillwater, OK, USA. *Ixodes pacificus* and *Ixodes ricinus* nymphs were provided by BEI Resources, CDC.

### 2.2. Artificial Feeding System Bloodmeal Optimization for B. burgdorferi Infection

Ticks were fed on either a bovine, bovine heat-activated, or rabbit blood mixture. Blood mixtures combined serum with red blood cells in a 9:1 ratio and supplemented with glucose. Blood receptacles were filled with 10 mLs of bovine blood or 8 mLs of rabbit blood. Bovine blood was provided by the USDA-ARS at Washington State University, Pullman, WA, and rabbit blood was obtained commercially (Innovative Research, Inc., Novi, MI, USA). Kanamycin was used to prevent bacterial contamination of bloodmeal.

Fresh bovine blood, heat-inactivated bovine blood, rabbit blood, and heat-inactivated bovine/rabbit blood hybrid were compared for potential borreliacidal effects. Fresh bovine serum was heat-inactivated at 53 °C for 90 min, and then recombined with red blood cells. The heat-inactivated bovine blood and rabbit blood hybrid were combined in a 1:1 ratio. B31-5A4 NP1 *B. burgdorferi* cultures were grown to mid-log phase and inoculated into 5 mLs of the compared blood cultures for the desired concentration of 32 spirochetes per 100 µL. After culturing overnight at 37 °C, 100 µL of each blood culture was inoculated into 5 mL BSK-II cultures for qualitative recovery analysis and separately inoculated 20 mLs of BSK-II, then plated onto 96-well plates for quantitative approximation of borreliacidal effects.

### 2.3. Recovering B. burgdorferi from Infected Ixodes Ticks

*Ixodes* ticks were infected over the duration of a bloodmeal from the artificial feeding system. Ticks were allowed 48 h to attach and feed on a pathogen-free bloodmeal at 37 °C. Blood was routinely replaced in 12 h intervals for fresh blood with kanamycin antibiotic at the final concentration of 200 µg/mL. After 48 h, the desired concentration of *B. burgdorferi* was directly inoculated into each bloodmeal. Ticks were observed to feed to repletion as early as day four post attachment. To allow replete ticks to drop off onto the fabric mesh, the *Ixodes* receptacle attachment is removed from the *Ixodes* adapter. Any replete ticks present in the receptacle were removed for storage and used cotton balls were discarded. Repleted ticks that fell off onto the fabric mesh were collected for storage. The remaining engorging ticks were allowed to feed until seven days post initial attachment, and were collected off the membrane using brushes and forceps and then stored.

Replete ticks were surface sterilized with 70% ethanol, patted dry with a paper towel, and then stored in a polystyrene test tube with a cell strainer snap cap. Ticks were stored at 26 °C at 94% humidity. Tick-containing tubes were housed in Tupperware containers with saturated potassium sulfate solution to maintain humidity.

After three, seven, or seventeen days of storage, ticks were surface sterilized in succession with 3% H_2_O_2_ for 2 min and 70% ethanol for 10 min, and then rinsed with DI water. Ticks were then submerged in individual 1.5 mL tubes with 0.5 mL of Barbour–Stonner–Kelly II (BSK-II) growth media containing *Borrelia* antibiotic cocktail (50 µg/mL rifampicin, 100 µg/mL fosfomycin, and 5 µg/mL amphotericin B). Ticks were then thoroughly crushed using a pestle and mortar. Additional BSK-II media with antibiotics were added to bring the final volume to 1.5 mLs and incubated at 35 °C under microaerobic conditions. Cultures were regularly checked daily for seven days using dark-field microscopy to confirm the presence of spirochetes. 

### 2.4. Bacterial Strains

The *B. burgdorferi* B31-5A4 NP1 strain, pIVET_Bb_ library pool of clones, 5A10::pIVET_Bb_-P_FlaB_ clone, and the 5A10::pIVET_Bb_ clone were previously described [13,22,23]. All *Borrelia* cultures were grown using BSK-II media supplemented with 6% rabbit serum, and kept in microaerobic conditions at 35 °C [24]. Bacterial growth and concentration were estimated using the Petroff–Hausser counting chamber and visualized using dark-field microscopy.

### 2.5. Recovering IVET B. burgdorferi Clones from Infected Ticks

Ticks were fed to repletion on infected bloodmeals, stored, surface sterilized, and then crushed in BSK-II growth media with antibiotics as previously described. The culture was recovered overnight and transferred into 1 mL of fresh BSK-II containing antibiotics to remove crushed tick parts. Cultures were filtered through 13 mm Syringe Filters with a 0.45 µm pore size (Acrodisc, Wilmington, DE, USA) to remove any potential contamination. Cultures were observed daily until viable spirochetes were observable through dark-field microscopy. Culture-positive and negative individual crushed ticks were then pooled into groups of ~5 ticks, inoculated into 100 mLs of BSK-II with antibiotics, and then plated into 96-well plates. After four days of incubation, the wells were replica-plated into 96-well plates with BSK-II media containing 100 µg/mL of gentamicin. Wells containing spirochetes only in the absence of gentamicin were picked for individual cultures.

### 2.6. PCR Analysis and Screening

Colonies present on plates were PCR screened and sequenced for the presence of *B. burgdorferi* gDNA fragment. Colonies were subcultured into 5 mLs of BSK-II media and grown to the late log phase. Total gDNA was extracted from the sub-cultures (Promega Wizard, Madison, WI, USA). The forward primer GTC AGG GCC GAG CCT ACA TGT GC and reverse primer AGT GCC AAG CTT GCA TGC CTG CAG were utilized for the colony screen and amplicon sequencing. Sequencing services and results of DNA samples were rendered by Eurofins Genomics LLC (Louisville, KY, USA). Sequence results were analyzed utilizing BLAST to find the gene associated with the promoter element.

### 2.7. qRT-PCR Analysis

Total RNA was extracted from strains grown in vitro to the late log phase using the RNeasy Mini kit (Qiagen, Venlo, The Netherlands). A Turbo DNase free kit (Invitrogen, Waltham, MA, USA) was used to remove any residual genomic DNA from RNA samples and reverse-transcribed to complementary DNA (cDNA) using a Superscript VILO cDNA synthesis kit (Invitrogen, Waltham, MA, USA), as per the manufacturer’s instructions. 

For quantitative expression of *Borrelia* genes in *I. scapularis*, ten ticks were pooled in two batches each, homogenized under liquid nitrogen, and suspended in TRIzol reagent (Invitrogen, Waltham, MA, USA). Total RNA was isolated following the manufacturer’s instructions using chloroform and precipitated using isopropanol. DNase treatment and cDNA synthesis were performed as described above. Quantitative PCR was performed using the QX200 Droplet Digital PCR (ddPCR) system (Bio-Rad Laboratories, Hercules, CA, USA), TaqMan probes, supermix for probes (no dUTP) including the QX200 Droplet Generator, the PX1 PCR Plate Sealer, and the QX200 Droplet Reader, all per the manufacturer’s instructions. PCR was performed on the C1000 Touch Thermal Cycler (Bio-Rad Laboratories) using the following cycling conditions: enzyme activation at 95 °C for 10 min, amplification for 40 cycles with a ramp rate of 2.0 °C/s for denaturation at 94 °C for 30 s and annealing and extension at 60 °C for 1 min with a ramp rate of 2.0 °C/s, enzyme deactivation at 98 °C for 10 min, and an optional hold at 4 °C. Values were expressed as relative copies of gene per *flaB* and calculated as an average ± standard error mean. For all qRT-PCR analyses, comparisons were made using an unpaired, two-tailed Student’s *t*-test. *p* < 0.05 was considered significant. Primers used for qRT-PCR are listed in Table S1.

## 3. Results

### 3.1. In Vitro Tick Feeding System Can Replace Mice as a Source of an Infected Bloodmeal for Ixodes *spp.* of Ticks

**Figure 1:**
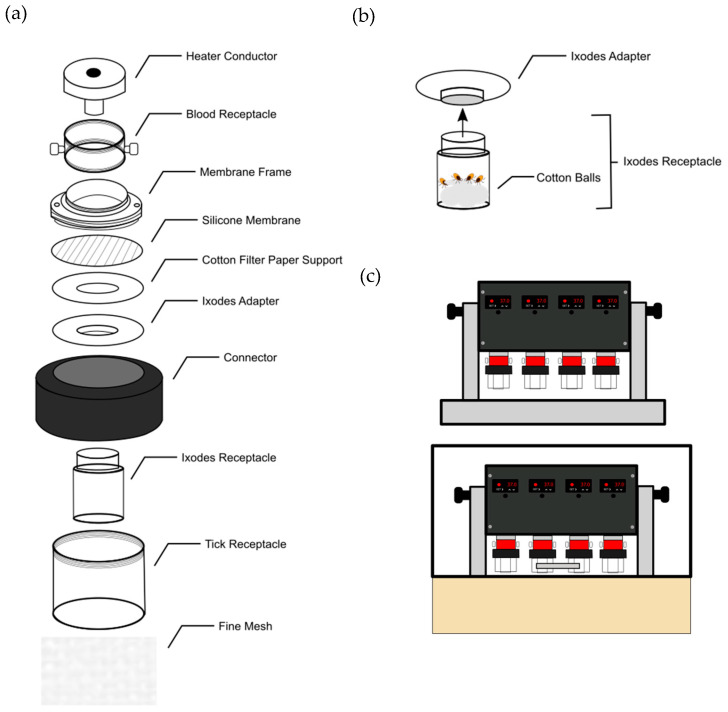
In vitro tick feeding system with *Ixodes* adapter. (**a**) Components of a tick feeding chamber optimized for *Ixodes* tick feeding. The assembly of the chamber is displayed in the sequence of the required components. The silicone membrane is 20 µm–40 µm thick and requires additional support and protection from a cotton filter paper cut out. The filter paper has a hole cut out at an identical circumference to the *Ixodes* adapter and is affixed to the surface of the membrane with additional silicone. An *Ixodes* adapter and receptacle have been developed to facilitate crowding and maintain humidity. (**b**) The attachment of the receptacle to the *Ixodes* adapter. The receptacle attaches to the adapter by slip fit for ease of removal. The bottom of the receptacle is stuffed with a layer of dry cotton balls to prevent tick escape, topped by a single damp cotton ball. Ticks are placed on top of cotton balls, then the receptacle is slipped onto the adapter. (**c**) The digital heater and humidity containment chamber. The digital heater controls the temperature the heat conductor reaches. One heater unit can attach up to four tick-feeding chambers at a time. The heating unit is contained in a chamber that can retain a humid environment.

A novel silicone membrane-based in vitro tick feeding system was recently described in Vimonish et al., 2020, for the feeding of *Dermacentor* spp. ticks [21]. This system was chosen and then modified for the feeding of *Ixodes* nymphs. The tick feeding system was designed to be easy to assemble, allow blood changes without disturbing tick attachment, and optionally operate with a peristaltic pump to circulate blood at a constant temperature that mimics living animals. For our study, we utilized the tick-feeding chamber and a digital heating power source. The original five-part feeding device was modified to include additional two parts to adapt it for *Ixodes* spp. ticks. The chamber is assembled by threads and the blood receptacle was designed to hold up to 20 mL (Figure 1a). As the initial feeding system was designed for use with *Dermacentor* spp. ticks, it required a number of modifications to account for the physiology of *Ixodes* spp. ticks. To accomplish this, the feeding system was altered to maintain a higher and consistent humidity, the silicon membrane thickness was reduced to 20–40 µm, and the overall containment and feeding area was reduced to encourage crowding feeding stimulus of ticks (Figure 1a–c).

To determine whether the modifications to the in vitro tick system would result in successful feeding and repletion of *Ixodes* ticks, groups of at least 50 *I. scapularis*, *I. pacificus*, and *I. ricinus* nymphal ticks were independently placed into the feeding system. At 48 hrs post attachment, *B. burgdorferi* 5A4 NP1 spirochete cultures were concentrated and inoculated into a bovine bloodmeal at a final concentration of either 5000 or 10,000 spirochetes per µL. The results showed that all tick species successfully attached with varying rates of repletion (Table 1). Ticks were allowed to feed for up to seven days, and engorged ticks were sterilized and crushed at days three, seven, and seventeen post-repletion for cultivation in BSK-II media to assess the presence of viable spirochetes. The recovered tick cultures found spirochetes visible throughout all three timepoints in *I. pacificus* and *I. ricinus*; however, no culture-viable spirochetes were observed from *I. scapularis* nymphs. This suggests that *Ixodes* ticks can successfully acquire spirochetes from the system, but susceptibility to *B. burgdorferi* colonization may vary between tick species or cohorts (Table 1). 

### 3.2. The Effects of the Bloodmeal Source on B. burgdorferi Infectivity of Ticks

After experimentally infecting *Ixodes* spp. ticks, we re-examined the controllable variables of *B. burgdorferi* infection of ticks via the in vitro feeding system. One possible factor affecting the survivability of the *B. burgdorferi* in *I. scapularis* ticks was the source of the bloodmeal (i.e., bovine versus rabbit), potentially due to differences in immune factors such as complement. To determine the potential influence of immune factors present in the bloodmeal, 5A4-NP1 spirochetes were inoculated into whole bovine blood, whole rabbit blood, heat-inactivated bovine blood, or mixed heat-inactivated bovine/rabbit blood at the desired concentration of 32 spirochetes per 100 µL of blood. After culturing overnight at 37 °C, 100 µL of each blood culture was separately plated onto 96-well plates. Whole bovine blood greatly diminished the survivability of *B. burgdorferi* spirochetes. In contrast, whole rabbit blood did not reduce from the expected recovery of viable spirochetes. Heat inactivation of the bovine serum led to improved *B. burgdorferi* survival, as did a heat-inactivated bovine/rabbit blood mixture (Table 2). 

After determining the most suitable bloodmeal for recovering viable *B. burgdorferi*, we assessed the ability of rabbit blood-containing spirochetes to infect *I. scapularis* ticks utilizing the in vitro feeding system. We repeated the previous experiment and demonstrated that using rabbit blood restores successful infection of *I. scapularis* ticks (Table 3).

### 3.3. Screening of a pIVET_Bb_ Promoter Library in I. scapularis Ticks

**Figure 2:**
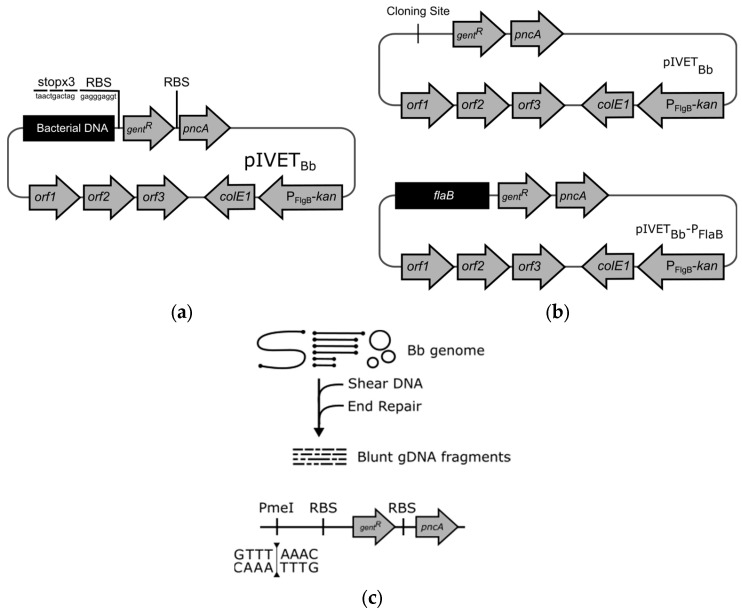
IVET reporter plasmid design. (**a**) Schematic for the pIVET_Bb_ reporter plasmid. The plasmid consists of in vitro and in vivo selection genes, *gent^R^* and *pncA*, respectively, that can be driven by a cloned bacterial DNA promoter fragment. The promoter fragments are cloned into a *pmeI* restriction site upstream of the selection genes. (**b**) The control strains of *B. burgdorferi* IVET screen. The negative control strain, 5A10::pIVET_Bb_, has no promoter in the cloning site. The positive control strain, 5A10::pIVET_Bb_−P_FlaB_, has a constitutive promoter of *flaB*. (**c**) The generation of the genomic DNA library of *B. burgdorferi* IVET system. The gDNA of *B. burgdorferi* was mechanically sheared, size-selected, end-repaired, and then cloned into the *pmeI* site upstream of the pIVET_Bb_ selection genes.

The successful implementation of the artificial feeding system made possible an IVET screen of a *B. burgdorferi* promoter library during tick infection. To do this, we chose to make use of our previously developed pIVET_Bb_ system [13]. Although this pIVET_Bb_ library was not originally optimized for selection within the tick vector, it was expected to restore some degree of infectivity within ticks due to the presence of the *pncA* gene as an in vivo selectable marker that has been previously shown to be important for infection of ticks by *B. burgdorferi* [13,19,25] (Figure 2a). The B31-5A10 clone of *B. burgdorferi* (5A10) lacks linear plasmid 25 (lp25), which harbors genes critical for the infection of *Ixodes* ticks, notably *pncA* [19,22,25,26]. In Casselli and Bankhead 2015, the negative control strain, 5A10::pIVET_Bb_, harbors a promoter-less *pncA* gene which is expected to be non-infectious in the tick (Figure 2b). The positive control strain, 5A10::pIVET_Bb_-P_FlaB_, harbors a constitutive *flaB* promoter driving *pncA* expression which is expected to restore infectivity, and also drives a gentamicin-resistance gene (*gent^R^*) for in vitro selection after isolation from the tick [13] (Figure 2b). To test the viability of the pIVET_Bb_ genetic screen, *I. scapularis* ticks were provided a bloodmeal containing either the positive or negative control strains. Repleted ticks were collected and allowed to rest seventeen days post-feed. The ticks were then pooled (4–6 nymphs/pool), surface sterilized, crushed, and then inoculated into BSK-II growth media. The cultures were observed daily until spirochetes were visible by dark-field microscopy. Once visible spirochetes were observed, they were subcultured into BSK-II culture supplemented with 100 µg/mL gentamicin.

The 5A10::IVET_Bb_-P_flaB_ positive control strain was recovered from cultures both with and without gentamicin. This result indicates that constitutively driven *pncA* permits in vivo colonization of nymphal ticks and would continue to drive *gent^R^* in vitro. Conversely, the 5A10::IVET_Bb_ negative control strain was not recovered from tick crush cultures, demonstrating an inability to infect and colonize ticks due to a lack of expression of the *pncA*.

**Figure 3:**
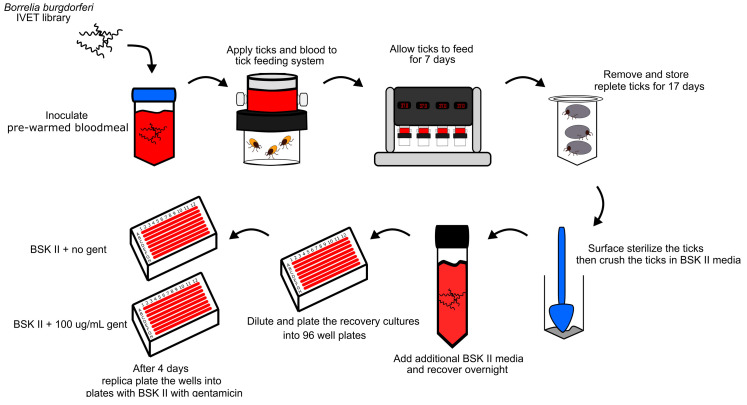
Workflow of IVET screen. *B. burgdorferi* B31-5A10 strain harboring the IVET promoter library is grown in vitro and inoculated into the bloodmeal of 50 nymphal *I. scapularis* ticks placed in an in vitro feeding system. Ticks acquire spirochetes via bloodmeal over seven days, then are removed from the feeding system for seventeen days of storage to allow in vivo selection for IVET clones harboring in vivo induced promoters. After seventeen days, ticks are cleaned, crushed, and then dilution plated for in vitro selection. Dilution plates are replica plated four days post-crush into BSK-II medium containing gentamicin to distinguish clones harboring tick-induced promoters.

To conduct the pIVET_Bb_ genetic screen, we utilized the pool of IVET clones that harbor gDNA fragments. The IVET pool was developed by mechanically shearing the gDNA of *B. burgdorferi* into 1–3 kbp fragments. The unique fragments were cloned into the *pmeI* restriction site, then the IVET plasmids were screened to ensure representation of the whole bacterial genome (Figure 2c). The pool of IVET plasmids was repeatedly transformed into *B. burgdorferi* 5A10 background strain, resulting in a polyclonal genomic library representing 6-fold bidirectional coverage of the bacterial genome [13]. We fed 50 nymphal *I. scapularis* ticks with bloodmeals containing the IVET pool. After 17 days post-repletion, the repleted ticks were surface-sterilized and individually crushed for overnight culture in BSK-II media. The overnight recovery cultures were dilution plated into five 96-well plates containing non-selective BSK-II media. After four days, the 96-well plates were replica plated into BSK-II with gentamicin selective media. The clones that harbor tick-induced promoters would grow in the non-selecting media exclusively. Clones with tick-induced promoters were then chosen for PCR amplification, DNA sequencing, and BLAST analysis to identify the associated genes (Figure 3). Tick crushes with observable viable spirochetes from the recovery culture were plated via limited dilution into 96-well plates without antibiotics and incubated for four days, followed by replica plating into BSK-II media containing gentamicin. The screen was able to identify a number of genes with no known tick function, as well as several genes previously associated with upregulated expression in ticks (Table 4). The *ospC* gene of *B. burgdorferi* is well-known to exhibit induced expression in ticks during feeding [27]. The *bb0400* gene was among a number of upregulated genes identified in an array of in vitro cultures exposed to blood and temperature shifts to mimic a tick-like environment [9]. The *bdr* gene family is present within all *Borrelia* species, and the expression of *bdr* paralogs was demonstrated to be specific to environmental changes within the tick [28]. 

### 3.4. Validation of Tick-Induced Gene Promoters Identified from IVET Screening

To validate the hits identified from the IVET promoter screen, relative transcription levels of the *B. burgdorferi* genes during tick infection were compared to expression levels in vitro via qRT-PCR analysis. *I. scapularis* nymphs were fed to repletion with bloodmeal inoculated with B31-5A4-NP1 wild-type *B. burgdorferi*. After incubating seven days post-repletion, the ticks were frozen and stored at −80 °C. Total RNA was extracted from crushed ticks and converted into cDNA. Total RNA was also isolated from in vitro-grown 5A4-NP1 spirochetes. 

**Figure 4:**
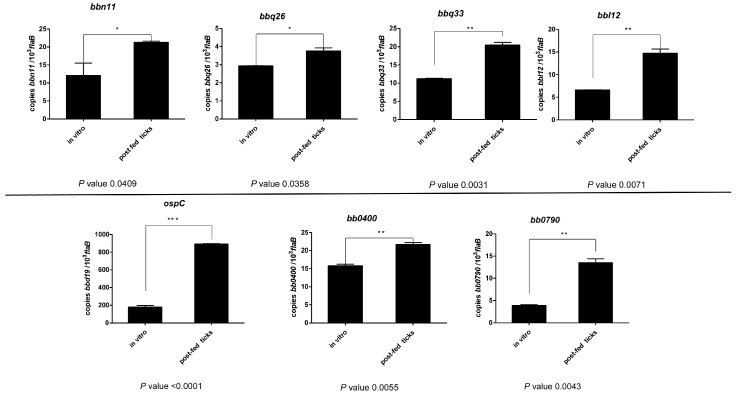
qRT-PCR analysis comparing the relative copies of cDNA per *flaB* between an in vivo tick infection of *B. burgdorferi* and in vitro culture. Values are expressed by averages ± standard error mean. Comparisons used a two-tailed Student’s *t*-test, and *p* < 0.05 was considered significant. *p < 0.05, **p < 0.005 and ***p < 0.0001.

Consistent with the IVET hits, transcription levels for seven of the eight genes were significantly increased in ticks compared to those in vitro, thereby validating the findings from the promoter screen (Figure 4). Similar to previously published studies, expression levels of *ospC* were highly upregulated in ticks, further validating our IVET screening method. Despite multiple attempts, we were unable to obtain detectable expression levels for the *bdrH* gene from both in vivo and in vitro samples. Overall, the results from the IVET screen and qRT-PCR analysis demonstrate the strong potential of our developed tick-feeding system for gene expression studies without the need for prior mouse infection.

## 4. Discussion

The IVET study in Casselli and Bankhead 2015 was hindered by a severe infection bottleneck in the mouse that prevented the wider application of the technology to arthropod vectors [13]. The present study aimed to overcome this technical challenge by utilizing an artificial tick-feeding system to perform the first *B. burgdorferi* IVET promoter screen in the *Ixodes* tick vector. To accomplish this, we adapted an in vitro feeding system to the principal vectors of *B. burgdorferi* and demonstrated its utility to infect and replete nymphal *Ixodes* spp. ticks. There are several physiological conditions to consider for *Ixodes* spp. ticks, including the length of the mouthparts to pierce and acquire a bloodmeal from the membrane and their susceptibility to desiccation while feeding. One other aspect we considered is the general vitality of the ticks. During the course of our study, we observed that nymphal ticks fed best when molted within a few months of the experiment. 

During our optimization, we found that the source and quality of the bloodmeal affect spirochete viability and infectivity. We observed that we were able to infect *I. pacificus* and *I. ricinus* using bovine blood, and unable to infect *I. scapularis*. We noted low spirochete titers and abnormal morphology from the *I. pacificus* and *I. ricinus* isolates. There could be a number of unknown factors between the *Ixodes* species of ticks to overcome the borreliacidal components of bovine blood, but to infect *I. scapularis* we pursued alternatives to potentially reduce or minimize immune components in the bloodmeal [29]. While heat inactivation improved spirochete survival in the bovine blood, we observed improved recovery in rabbit blood. The rabbit blood restored the *Borrelia* infection of *I. scapularis* nymphs, but we found that the blood declined in quality much faster once on the heating element despite the presence of antibiotics. In addition, the rabbit blood was noticeably thinner than the bovine mixture which would cause the membrane to leak from microtears caused either by mechanical abrasions or ticks. It is uncertain whether this is due to the difference in methods by which our lab in-house processes fresh bovine blood versus the supplier’s process with rabbit blood, or an innate quality of blood between the two animals.

Our IVET screen provided proof of principle for the potential of utilizing a promoter trap system with our developed tick feeding unit. To acquire a full list of genes induced in the tick environment would likely require a number of tick feedings to ensure all *B. burgdorferi* library clones would be initially acquired by the feeding ticks. Our IVET screen led to the identification of promoters of genes previously undocumented to have a function within a tick, and successfully identified a gene that is well-known to be highly induced in feeding ticks [27]. The findings were further validated by qRT-PCR analysis, which demonstrated relatively higher expression levels of the IVET-identified genes during tick infection compared to in vitro. Together, these findings suggest that given additional tick feedings and screens, the likelihood is that the IVET system would find additional genes that are induced in a tick environment.

While we have demonstrated that an IVET system screen is feasible in *I. scapularis*, several technical hurdles are needed for a complete screen. As previously discussed, *B. burgdorferi* has a linear plasmid, lp25, that is essential for tick infection [19,20]. The role of the different genetic elements on lp25 for a tick infection is relatively understudied; however, previous examinations found that *pncA* and *bptA* are two of several unknown genetic components for the effective colonization of the tick vector. It is understood that the nicotinamidase gene, *pncA*, is essential for both infection of the mammal and the tick; however, restoration of *pncA* alone in a lp25 deficient background only minimally restores infectivity relative to a wild type [19,20,25,30]. Further studies identified and determined the role of the *bptA* gene, which is another component for the sustained infection of the tick and enhances infection of mammals, but again *pncA* and *bptA* together can only partially restore infectivity [21,22]. These two genes provided a promising target to function as an in vivo selection gene for an IVET reporter plasmid for ticks, but further study of the genetic elements of this pathogen may yield a better candidate. In addition, there is a disadvantage in the use of a 5A10 background strain in the design for an IVET system, as this strain lacks the 56 kb linear plasmid, lp56. The role of lp56 is understudied in a tick infection; while non-essential for a tick infection, the absence of the plasmid significantly reduces the normal expansion of the bacteria in the tick [31]. Future design of a tick-specific IVET system would ideally avoid background strains missing linear plasmids carrying several virulence factors in ticks. 

While the disadvantages were initially considered, our hope was that the overall advantages associated with the in vitro tick feeding system would overcome the challenges associated with performing the IVET screen. The in vitro tick feeding system permits direct inoculation into the bloodmeal to control the desired spirochete density the tick ingests compared to the traditional infected mouse feeding. If the bloodmeal has a sufficient density of spirochetes, there is potential to overcome the decreased bacterial survival in the tick. Additionally, unlike the IVET studies performed on mice, we are less constrained by the number of hosts used in this study. Due to the severe infection bottleneck, prior IVET studies could have likely identified additional host-induced promoters of *B. burgdorferi* if provided the unrealistic opportunity of using large numbers of mice [12,13]. An additional benefit of the IVET system is the capacity to perform the screen on a sufficiently large quantity of ticks per chamber. Once an average amount of spirochetes acquired from a feeding per tick is established, the number of ticks needed for a whole genome screen can be determined as needed. Should a genome screen require many ticks, the chamber can be scaled up to accommodate a large group.

The results obtained from this study demonstrate the utility of the in vitro tick feeding system and proof of principle for the IVET genetic screen. The development of the in vitro tick feeding system provides another tool to control the bloodmeal for the *Ixodes* spp. ticks. In addition to the benefit of replacing the use of rodents, the in vitro tick feeding system gives additional control over tick experiments by determining the titer of inoculated infectious bacteria as well as desired compounds in the bloodmeal. Despite the restoration of only partial infectivity of the IVET system relative to a wild-type clone of *B. burgdorferi*, we were able to show the feasibility of this screen by yielding a number of promoters that are induced in the tick environment. While a handful of technical challenges remain for a complete IVET screen, a genome-wide screen of *B. burgdorferi* tick-associated genetic elements is a necessary component to understand the continued propagation of this emerging pathogen. The method by which *B. burgdorferi* adapts to a tick is understudied and an IVET screen can identify genes that are essential to the process. Targeting these essential genes has the potential to disrupt the enzootic cycle or prevent human infection. *B. burgdorferi* is a challenging bacterium to study and many of our current methods to prevent infection are still targeting limited genes identified decades ago. As additional studies are performed on commonly identified tick-associated genes, more effective alternatives can be pursued.

## Figures and Tables

**Table 1:** Attachment, repletion, and infection levels of *I. scapularis*, *I. pacificus*, and *I. ricinus* ticks fed with a bovine bloodmeal containing *B. burgdorferi*.

Tick Species	Spirochete Density (Spirochetes/µL)	Attachment (%)	Engorgement (%)	Infected 3 Days Post-Repletion (%) ^a^	Infected 7 Days Post-Repletion (%)	Infected 17 Days Post-Repletion (%)
*I. scapularis*	5000	(46/50) 92%	(33/46) 71.7%	(0/11) 0%	(0/11) 0%	(0/11) 0%
*I. scapularis*	5000	(35/50) 70%	(30/35) 85.7%	(0/10) 0%	(0/10) 0%	(0/10) 0%
*I. scapularis*	10,000	(37/50) 74%	(33/37) 89.2%	(0/11) 0%	(0/11) 0%	(0/11) 0%
*I. scapularis*	10,000	(36/50) 72%	(26/36) 72.2%	(0/8) 0%	(0/9) 0%	(0/9) 0%
*I. pacificus*	5000	(12/50) 24%	(8/12) 66.7%	(0/2) 0%	(2/3) 66.7%	(1/3) 33.3%
*I. pacificus*	10,000	(14/50) 28%	(9/14) 64.3%	(2/3) 66.7%	(2/3) 66.7%	(1/3) 33.3%
*I. ricinus*	5000	(42/50) 84%	(39/42) 92.9%	(4/13) 30.8%	(5/13) 38.5%	(3/13) 23%
*I. ricinus*	10,000	(40/50) 80%	(36/40) 90%	(10/12) 83.3%	(4/12) 33.3%	(3/12) 25%

^a^ Cultures of individual crushed ticks were determined as infected when live spirochetes were visible under dark-field microscopy.

**Table 2:** *B*. *burgdorferi* viability in bovine and rabbit blood.

Blood Type	Dilution Plate, Positive Wells
Bovine	(0/96)
Rabbit	(32/96)
Bovine, Heat Inactivated Serum	(16/96)
Rabbit/Bovine Heat Inactivated Serum	(15/96)

Wells of 96-well plates were determined as positive when live spirochetes were observed under dark-field microscopy.

**Table 3:** *Ixodes scapularis* ticks feeding, repleting, and acquiring *Borrelia* spirochetes inoculation of in vitro tick system with rabbit blood.

Tick Species	Spirochete Density (Spirochetes/µL)	Attachment (%)	Engorgement (%)	Infected 3 Days Post-Repletion (%) ^a^	Infected 7 Days Post-Repletion (%)	Infected 17 Days Post-Repletion (%)
*I. scapularis*	2000	(29/50) 58%	(23/29) 79.3%	(2/7) 28.6%	(1/7) 14.3%	(2/8) 25%

^a^ Cultures of individual ticks were determined as infected when live spirochetes were visible under dark-field microscopy.

**Table 4:** IVET screen results from infected *I. scapularis* nymphal ticks.

Gene Name	Annotated Function	Known Tick Association	Reference
*bbn11*	Hypothetical Protein	No	-
*bbq26*	Hypothetical Protein	No	-
*bbq33*	D family	No	-
*bbl12*	Hypothetical Protein	No	-
*ospC*	Lipoprotein	Yes	[27]
*bb0400*	Hypothetical Protein	Yes	[9]
*bdrH*	Transmembrane P	Yes	[28]
*bb0790*	Hypothetical Protein	No	-

## Data Availability

The original contributions presented in the study are included in the article/Supplementary Material, further inquiries can be directed to the corresponding author.

## Supplementary Materials

The following supporting information can be downloaded at: https://www.mdpi.com/article/10.3390/pathogens13060487/s1, Table S1: IVET Hits Primers and Probes.
