# Supplementary material for: Development and Application of an In Vitro Tick Feeding System to Identify Ixodes Tick Environment-Induced Genes of the Lyme Disease Agent, Borrelia burgdorferi
Source: Pathogens. 2024 Jun 7;13(6):487. doi: 10.3390/pathogens13060487 (PMC11207009; doi:10.3390/pathogens13060487)
Supplement: Supplementary file 1 [file pathogens-13-00487-s001.zip › Table S1 IVET_final.pdf]

Table S1: IVET Hits Primers and Probes

1. ***bb_n11***

N11F- AAGTCAATATCTCCATCAAGC
N11R- TTGCAGTTCGCAACTTTCC
N11 probe- HEX-TGCCGCTGGCTGCTACACCA-BHQ1

2. ***bb_q26***

Q26F- GTAGTTTAAGCTATGCTCCTC
Q26R- ATTCAAAACATCGCCTGAG
Q26probe- HEX-ACAAGACAACAAAGCACACATATCCT-BHQ1

3. ***bb_q33***

Q33F- CATTGTCTACTAATTGCTTTGC
Q33R- TAGTGTTGGAATAATTGGCG
Q33 probe- HEX-TGCACCACTATTGCCAACCTACCAGA-BHQ1

4. ***bb_l12***

L12F- TTCAATTGGTGGTACGCAGTTAC
L12R- GGATCTCTTAAGCTTATTACAGGC
L12 probe- HEX-TGGCAAGCTAGAGCTTACAAGCGAACCTAC-BHQ1

5. ***bb_b19* (*ospC*)**

B19F- CGGATTCTAATGCGGTTTTAC
B19R- CCGCTAACAATGATCCATTGTG
Probe- HEX-CTATAGATGACAGCAACGCTTCAACCTCTT-BHQ1

6. ***bdrH* (*bb_r27*)**

R27F- AGTGTCAACAAATATTGCAG
R27R- CTCTATATGTTAGTTCATTGTGA
R27 probe- HEX-CATGGAACAACTAATAGCACAAGATTTATC-BHQ1

7. ***bb_0400***

0400F- TGAAGTTATCAGAAGCGCCAA
0400R- TCGATTTCCCAAACAAGCTG
0400 probe- HEX- TGCCTCTTGGCCCTATCGCAAGT-BHQ1

8. ***bb_0790***

0790F- ACCTATATGCAATTTCAGTCAAAG
0790R- TCCGTTAAAGCAACATTCTCTT
0790 probe- HEX-TTGCTAGCTTCAGATGCTCTTTATGAGCTT-BHQ1

9. ***bb_c12***

C12F- GCTCAACAATTGATGGCTGG

C12R- ACAATACTCAAGCCCAGTGC
C12probe- HEX-ACAAGCACAAGTGCAGCTAGCGT-BHQ1

10. ***flaB***

P199 - TTG CTG ATC AAGCTC AAT ATA ACC A
P200 - TTG AGA CCC TGAAAG TGA TGC
P201- 6FAM-CAGCTGAAGAGCTTGGAATGCAGCCT-TAMRA
